# Interventions for stigma reduction in HIV treatment and prevention designed to enhance antiretroviral uptake and adherence: A systematic review

**DOI:** 10.1371/journal.pgph.0004604

**Published:** 2025-05-12

**Authors:** Amit “Mickey” Dhir, Amteshwar Singh, Sunil Solomon, Jason E. Farley

**Affiliations:** 1 Johns Hopkins University School of Nursing, Baltimore, Maryland, United States of America; 2 Department of Medicine, Johns Hopkins University School of Medicine, Baltimore, Maryland, United States of America; McGill University, CANADA

## Abstract

HIV remains a significant public health burden worldwide, especially in developing countries, despite the availability of effective prevention and treatment modalities, namely PrEP and ART, respectively. HIV-related stigma is a significant barrier to the optimal uptake or adherence to ART or PrEP in vulnerable populations. Therefore, there is a need for effective interventions that can address HIV-related stigma and improve adherence to ART or PrEP in vulnerable populations. This systematic review aimed to identify interventions that can effectively reduce HIV-related stigma and improve adherence to ART or PrEP in vulnerable individuals. A systematic review methodology following the PRISMA guidelines was used. The sources of information included MEDLINE (via PubMed search engine), Embase, Scopus, Web of Science, CINAHL, and PsycINFO. Studies were eligible if they were randomized controlled trials (RCTs) or quasi-experimental studies investigating the effectiveness of any intervention addressing HIV-related stigma among persons living with HIV and improving ART/PrEP adherence. We adopted a narrative synthesis approach to present our findings. For quality appraisal of included studies, we used Cochrane Risk of Bias (RoB 2) tool.Eight RCTs, but no quasi-experimental studies, met the eligibility criteria. Among the seven RCTs on ART adherence, four investigated the effectiveness of cognitive behavioral components (two studies showing positive impact on stigma reduction and ART adherence). Other effective interventions that improved ART adherence while reducing HIV-related stigma included empowerment program and youth peer-mentoring. Only one study focused on PrEP (comprising motivational interviewing, client-centered counseling), which showed stigma reduction and PrEP adherence. Although many interventions have the promise of HIV stigma reduction while simultaneously improving medication adherence, larger scale studies are needed for generalizability. The heterogeneity in measurement instruments for ART/PrEP adherence underscores the need for standardized scales. Cognitive behavioral therapy, empowerment interventions, youth peer-mentoring, motivational interviewing, and client-centered counseling all show effectiveness in reducing HIV self-stigma, while simultaneously improving medication adherence. Future studies should consider the inclusion of diverse populations and refining interventions to address HIV- stigma, especially about PrEP. It is critical to incorporate validated measurement tools to enhance comparability across research endeavors to address the complex interplay between stigma and adherence. **PROSPERO number**: CRD42023455610.

## Introduction

The persistent global burden of the human immunodeficiency virus (HIV) continues to pose a significant public health concern, with 1.3 million new infections reported in 2022 [1]. While a cure remains elusive, HIV antiretroviral therapy (ART) treatment and Pre-exposure prophylaxis (PrEP) offer a significant solution by containing HIV transmission and promoting longevity among persons living with HIV (PLWH) [2–5]. Although cost-effective and efficacious, the global ART utilization rate remains suboptimal; out of the 39 million PLWH, only 29.8 million received treatment in 2022 (76.41%) [6]. Similarly, despite the profound benefits of PrEP, its global uptake remains quite dismal [2]. The United States(U.S.), for instance, has a mere 19.9% PrEP adoption rate among eligible men who have sex with men (MSM) with low long-term adherence rates [7,8]. A major reason for this low utilization is the pervasive HIV-related stigma. HIV-related stigma poses a formidable barrier to ART and PrEP adherence, which continues to threaten the efficacy of programs designed to end the HIV epidemic [9,10]. So much so that the Joint United Nations program for HIV/AIDS (UNIADS) and World Health Organization (WHO) have flagged the problem of stigma as a critical obstacle in HIV/AIDS management [11–13].

Goffman describes stigma as “*an attribute that is deeply discrediting*” [14]. Within the context of HIV, stigma is theorized to stem from mechanisms at play within both PLWH and HIV-negative individuals [15]. HIV-related stigma finds its origins in the fear and adverse public reactions during the initial AIDS epidemic [16]. HIV-related stigma is conceptualized as societal stigma (directed from society to HIV-positive individuals) and self-stigma (the negative feelings toward oneself as a member of a stigmatized group) [17]. A study by Baugher et. al. found that 79.1% (n = 13,841; 95% CI 77.4–80.7) of people living with HIV receiving medical care reported experiencing at least one form of stigma. The Medical Monitoring Project, which examines the characteristics of U.S. adults living with HIV who are in care, through a national probability sample, utilized weighted data collected between June 2011 and May 2014 to assess self-reported internalized stigma based on agreement with six statements. However, there was variability in the experiences of stigma based on race, gender, and socio-demographic factors. This study notes highest stigma rates among Hispanic/Latina females and the lowest among white males [18]. The damaging consequences of HIV stigma include low patient engagement, fear of disclosure, substandard healthcare-seeking behaviors, interpersonal strain, social adjustment challenges, social isolation, professional disengagement, concerns about family planning or pregnancy, withdrawal from education, and mental health challenges [19,20]. These further contribute to reduced adherence rates, creating a vicious cycle for the patients [9,21]. HIV-related stigma causes loss of social support and adaptive coping [22]. In addition, stigma dissuades individuals from disclosing their HIV status to family, friends, and sexual partners. This creates isolation, making it challenging to access and adhere to treatment, seek counseling, and use transmission-prevention strategies such as condoms, PrEP, Doxycycline Post-Exposure Prophylaxis (Doxy-PEP), etc. [23].

To address the negative impact of HIV stigma, the research on this subject is gaining momentum; the number of NIH-funded projects investigating HIV stigma has doubled between 2015 and 2019 [24]. Healthcare professionals, health systems, policymakers, lawmakers, and pharmaceutical stakeholders must recognize the consequences of HIV-related stigma. Equally important is to design and implement targeted interventions for bolstering adherence to ART/PrEP [25]. However, there is a dearth of evidence on the effectiveness of HIV-stigma reduction interventions [26]. Existing interventions have demonstrated the potential for reducing stigma, but few have simultaneously addressed both stigma and adherence [27].

We designed this systematic review to assess interventions addressing HIV-related stigma, defined as either HIV- or PrEP-related stigma, and the association with ART or PrEP adherence. The objectives of this study include identifying key factors influencing successful stigma reduction, elucidating the mechanisms that link stigma-reduction interventions with adherence outcomes, and describing gaps in the literature on interventions that target both stigma reduction and ART/PrEP adherence.

## Methods

### Research design and search strategy

This systematic review adheres to the Cochrane Collaboration standards [28]. We used the Preferred Reporting Items for Systematic Reviews and Meta-analyses (PRISMA) guidelines to report our findings [29]. We included randomized controlled trials (RCT), or quasi-experimental studies published between January 1, 2013, and June 1, 2023. We repeated the search in May 2024 to identify any new studies. We registered this systematic review with PROSPERO (CRD42023455610). No protocol was published or amended.

We employed a comprehensive strategy to search six electronic databases: MEDLINE (PubMed), Embase, Scopus, Web of Science, CINAHL, and PsycINFO [30]. We also conducted supplementary searches through manual methods, including hand and bibliographic searches [31]. The review period was June 2023 to March 2024. We wrote the manuscript between March 2024, and July 2024.

The search strategy involved a meticulous breakdown of the research topic into three conceptual units: stigma, HIV, and adherence. Medical Subject Headings (MeSH) terms were identified for each unit, further enhancing the search efficacy within the MEDLINE database through PubMed [32]. The MeSH terms selected were: “Stigma, social stigma, internaliz(s)ed stigma and stigma reduction” [Mesh] for stigma and stigma reduction, “HIV” [Mesh], “Antiretroviral Therapy, Highly Active, ART, therapeutics” [Mesh], and “Pre-Exposure Prophylaxis, PrEP” [Mesh] for HIV, and “Medication Adherence, compliance, uptake” [Mesh] for adherence and uptake. We combined the MeSH terms with relevant keywords using Boolean connectors (AND & OR) [33]. Tailored truncators and field tags were deployed for each database, targeting titles, titles/abstracts, and full texts to improve search precision (Fatehi et al., 2014). The search strategy development was peer-reviewed by an information specialist based on the Peer Review of Electronic Search Strategies (PRESS) checklist [34]. The strategy is available in S1 File.

### Study eligibility and selection process

Inclusion criteria encompassed studies published within the last decade (2013–2023) in the English language, peer-reviewed, and with accessible full-text versions. The Population-Intervention-Comparison-Outcome (PICO) framework guided the development of additional eligibility criteria [35]. Studies were eligible if the participants were 15 years old and older, either persons living with HIV on ART or at-risk populations taking PrEP. We included studies if the interventions addressed HIV- or PrEP-related stigma (intervention) and reported outcomes related to stigma reduction, ART or PrEP uptake, or adherence. Studies that addressed only stigma reduction or medication adherence but not both were not included, based on the predefined search strategy. We included only experimental RCTs or quasi-experimental studies assessing the intervention’s impact on the targeted outcomes. Exclusion criteria comprised studies with unavailable full texts, interventions not tailored to enhance ART/PrEP compliance or uptake, and lack of reported effectiveness in improving ART/PrEP adherence/uptake or stigma reduction. We also excluded studies that reported only stigma reduction without any outcome measurement for ART/PrEP adherence/uptake.

We used the online software Covidence to manage literature search. We removed duplicate entries after initial eligibility screenings based on abstracts and titles. We then evaluated the shortlisted studies. We resolved disagreements between reviewers (A.D., A.S.) through discussion to reach a consensus.

### Data extraction and synthesis

Two independent reviewers (A.D., A.S.) extracted essential data points from the articles: study particulars (authors, publication year, location, study design, follow-up period, participant characteristics, and sample size), stigma-related information (stigma type, assessment method), stigma reduction interventions (intervention type, description, effectiveness), and outcomes (operationalization of ART/PrEP adherence/uptake, assessment tool used, reported results). Two authors A.D. and A.S. met to discuss the extracted data points to summarize the methodology and results of each study. These discussion notes were then used by the author A.D. to draft the tabulated findings and the manuscript. We presented the data for each study in a structured tabular format. Disagreement was settled through a collaborative discussion.

Due to notable heterogeneity in participant characteristics, intervention attributes, and measurement tools, we performed a separate synthesis for RCTs and quasi-experimental studies, aligning with recommendations from Cochrane Consumers and Communication Review Group (2013) [36]. We adopted a narrative synthesis approach, comparing disparities and commonalities in findings and contributory factors, including study design and implementation. This helped identify stigma reduction interventions that enhance ART/PrEP adherence, delineating their underlying mechanisms and pinpointing areas requiring research for deeper insights.

### Quality assessment of the articles

A.D. and A.S. independently conducted and documented a rigorous quality assessment. In quasi-experimental studies, we assessed the risk of bias using the Cochrane Collaboration’s risk of bias assessment tool for non-randomized interventions [28]. To evaluate the risk of bias in RCTs, we used the Cochrane Risk of Bias (RoB 2) tool [37]. RoB2 has five domains: Domain 1(a) concerns bias arising from the randomization process, and 1(b) concerns bias arising from the recruitment of a cluster-randomized study (applicable to one study). Domain 2(a) assesses bias arising from the effect of assignment to intervention and 2(b) explores the effect of adhering to intervention. Domain 3 assesses bias due to missing outcome data; Domain 4 investigates bias in measuring the outcome; and Domain 5 is the arising from selecting the reported result. “High risk of bias” in any single domain assigns an overall high risk to the RCT.

## Results

**Fig 1:**
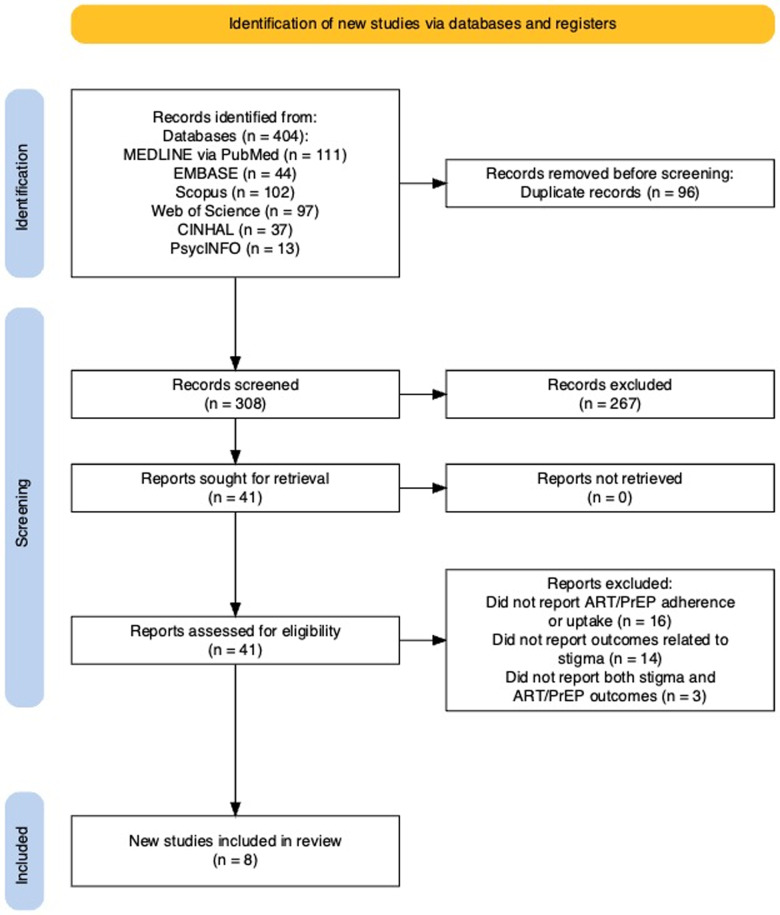
PRISMA flowchart showing study selection process [29].

All the predetermined sources of information yielded 404 records; we removed 96 duplicate records. We screened the remaining 308 records for eligibility based on titles and abstracts. After removing 267 records, we screened the full texts of the remaining 41 manuscripts. Reasons for exclusion at this stage included unreported outcomes related to ART/PrEP adherence despite reporting HIV-related stigma changes [38–42], and unreported outcomes related to both stigma and adherence due to qualitative approaches [43] (See S2 File). Eight RCTs met the eligibility criteria; seven focusing on ART [44–50] and one on PrEP [51] (Fig 1). No quasi-experimental or non-randomized controlled trials met inclusion criteria.

All seven RCTs focusing on improved ART uptake/adherence had two-arm parallel design. The singular PrEP-focused study by Eaton et al. (2023) employed a four-arm parallel design [51]. The studies were conducted in six countries: U.S. [46,47,51], South Africa [49], Zambia [48], Uganda [50], Pakistan [44], and Nepal [45]. [52] All participants were recruited from HIV treatment clinics, except Eaton et al. (2023), who recruited their participants via social media and snowballing. All studies used clearly defined inclusion/exclusion criteria. Participants’ age ranged from 15 to 55 including 534 males and 523 females; the studies conducted in the U.S. primarily focused on ethnic and gender minorities (Table 1).

**Table 1:** Participant characteristics of all the studies eligible for inclusion published between January 1, 2013 to June 1, 2023 (data extracted from June 2023 to March 2024).

Citation	Participant Age	Sample Size		Race and Ethnicity	Sex	
		Experimental	Control		Male	Female
Abbas et al. (2023) [44]	Range of 20–55 years. Mean age 31.87 (experimental, SD 8.35), Mean age 31.93 (control, SD 8.53)	63	63	NR	52	74
Bhatta and Liabsuetrakul (2017) [45]	18 years and above. Mean age 36.1 years (SD = 7.8)	66	66	non-indigenous (n = 74), Indigenous (n = 58)	62	70
Bogart et al. (2021) [46]	18 years and above. Mean age 52.9 years (SD = 12.9)	38	38	Identifying as Hispanic or Latino	76	0
Bryant et al. (2023) [47]	Range of 18–50 years. Mean age of 32.15 years (SD = 7.21)	66	64	Identifying as Black, cis-gender women	0	130
Denison et al. (2020) [48]	Range of 15–24 years. Mean age of 19 years	137	136	NR	111	162
Kalichman et al. (2019) [49]	18 years and above. Mean age of 34 years	25	25	NR	13	37
Wagner et al. (2021) [50]	18 years and above. Mean age 35.5 years (SD = 10.5)	49	44	NR	43	50
Eaton et al. (2023) [51]	18 years and above (36% below age 30, 64% above)	Jumpstart only – 51; Jumpstart text – 50; Jumpstart call – 51.	25	Identifying as Black	177	0

All seven ART studies intentionally enrolled individuals already diagnosed as HIV-positive and receiving ART, verified through meticulous examination of medical records. This deliberate focus on individuals actively engaged in HIV treatment aligns with the studies’ shared objective of evaluating interventions within a population already navigating the complexities of ART. While some studies delineated specific criteria for the duration of ART treatment inclusion, the majority did not impose such constraints, allowing for a diverse representation of individuals at various stages of their treatment. This nuanced approach acknowledges the heterogeneity within the HIV-positive population and captures the varied experiences and challenges associated with sustained adherence to antiretroviral regimens.

Eaton et al. (2023) included HIV-negative individuals in the PrEP investigation study [51]. In addition, this study included individuals who were unaware of their HIV status, who did not currently use PrEP but had engaged in condomless anal sex within the past year. The inclusion criteria across all studies showcase a strategic diversification, acknowledging the importance of addressing adherence and uptake concerns across different risk profiles within the broader context of HIV and HIV prevention.

Across the spectrum of these studies, a coherent pattern emerges in the exclusion criteria, reflecting a meticulous approach to participant selection. The criteria collectively aimed to ensure a cohesive and representative sample while minimizing confounding factors that could impact the study’s outcomes. First, a shared criterion involved participants’ language proficiency, emphasizing comprehension and reading ability. This criterion was often applied to both English and native African languages, underscoring the significance of effective communication and understanding in the research process. Second, the exclusion of individuals with physical and psychiatric comorbidities indicates a deliberate effort to isolate the impact of the stigma reduction interventions on HIV-related stigma and adherence. By excluding individuals with additional health challenges, the studies sought to maintain a focus on the specific interplay between the interventions and the targeted outcomes. Third, the requirement for participants to be available throughout the study period suggests a commitment to longitudinal engagement. This criterion likely aimed to mitigate potential bias introduced by participant dropouts, ensuring a more robust evaluation of the intervention’s sustained effects over time. Lastly, a notable exclusion criterion was the previous participation in an intervention with similar effects, specifically, the reduction of HIV-related stigma and enhancement of ART/PrEP adherence. This precautionary measure aimed to minimize the influence of prior interventions on participants’ responses, allowing for a more accurate assessment of the specific intervention under investigation.

**Table 2** provides a comprehensive overview of the inclusion and exclusion criteria employed across the eight randomized controlled trials (RCTs), highlighting the shared considerations that guided participant selection and reinforced the methodological rigor of these studies.

**Table 2:** Study characteristics of all the studies eligible for inclusion published between January 1, 2013 to June 1, 2023 (data extracted from June 2023 to March 2024).

Citation	Study Design	Country	Follow-Up	Participant Characteristics		Stigma-Related Data	
				Inclusion	Exclusion	Type	Assessment Method
Abbas et al. (2023)	Open-label, two-arm, parallel design RCT	Pakistan	Not stated	Diagnosed with HIV/AIDS; receiving ART regularly; mean score of > 5 in PHQ-9;	Pregnancy; substance use disorder; physiological or psychiatric comorbidity.	Social stigma	40-item HSS
Bhatta and Liabsuetrakul (2017)	Open-label, two-arm, parallel design RCT	Nepal	3 months; 6 months	Diagnosed with HIV/AIDS; receiving ART between 6 months and 2 years prior to the study	Severe health comorbidities; attended similar interventions in the past; unable to attend all follow-up visits; unwilling to disclose HIV status.	Internalized stigma	23-item questionnaire
Bogart et al. (2021)	Pilot, open-label, two-arm, parallel design RCT	United States	4 months; 7 months	HIV positive; have had sex with another man; not currently taking ART or missed ART at least once in the previous month.	Transgender individuals	Internalized stigma	Internalized AIDS-Related Stigma Scale
Bryant et al. (2023)	Two-arm, open-label, parallel design RCT	United States	Not stated	Living with HIV; residing in the Southern US; able to read and comprehend English.	Previous participation in the intervention;	Internalized stigma	Negative Self-Image Scale
Denison et al. (2020)	Two-arm, single-blind, parallel design RCT	Zambia	6 months	Aware of HIV status; been on ART for 6 months or more; spoke English or Bemba; available for study activities for the next 18 months	Too sick to participate; attending boarding school; having a sibling already enrolled; having participated in another intervention held at the study sites.	Internalized stigma	Internalized AIDS-Related Stigma Scale
Kalichman et al. (2019)	Two-arm, double-blind, parallel design RCT	South Africa	2 weeks	HIV-positive; currently receiving combined ART; unsuppressed HIV viral load; have a cellphone.	Currently in an adherence club, suppressed HIV viral load	Stigma-related medication management	3-item questionnaire
Wagner et al. (2021)	Clustered, open-label, two-arm, parallel-group RCT	Uganda	5 months; 8 months	Under HIV care for at least 1 year; having disclosed HIV status to more than 1 person.	Not explicitly stated	Internalized stigma	Internalized AIDS-Related Stigma Scale
Eaton et al. (2023)	Four-arm, open-label, parallel-group RCT (1:2:2:2)	United States	1 month; 2 months; 4 months	HIV negative/unknown status; condomless anal sex in the past year; HIV testing was more than 3 months ago; no current PrEP use	Not explicitly stated	Anticipated PrEP stigma	3-item scale

HSS = HIV Stigma Scale; PHQ-9 = Patient Health Questionnaire.

### Interventions for ART adherence

Four of the seven studies that focused on interventions intended for improved ART adherence comprised a cognitive-behavioral therapy (CBT) component delivered alongside other psychological treatment approaches, unique for each study [44,46,49,50]. Due to the unique combinations with cognitive behavioral strategies, the activities implemented in each of the four studies differed with a few areas of similarity. The most common activities implemented in the four studies included psychoeducation, skills training (e.g., coping skills), and motivational interviewing [44,46,49,50]. Abbas et al implemented 8-session blend of classic and brief CBT (b-CBT) activities to each participant individually to enhance motivation, manage stress, and provide cognitive restructuring for relapse reduction [44]. Bogart et al employed an intervention named *Siempre Seguiré* (Spanish for “I will always continue”), a blend of CBT and dialectical behavioral therapy (DBT) in 8 group sessions, aimed to enhance coping using intersectionality perspective, i.e., whole identity of a person, which is not just defined by their diagnosis [44,46]. In contrast, Kalichman et al employed five weekly-delivered phone-based counseling sessions for behavioral self-regulation, whereby participants would develop problem solving strategies to self-identified barriers to HIV care [49]. Wagner et al used compassion-focused therapeutic principles and CBT in six group sessions grounded in self-efficacy development through experience-sharing, support building, group problem solving, and personal goal setting [50]. While quite heterogenous, all four studies used CBT as the core intervention to enhance adherence.

Bhatta et al. implemented an empowerment intervention derived from social learning and action theories, comprising of group discussion sessions over 6 weeks [45]. Two national-level trainers with public health graduate degrees facilitated all intervention sessions, employing participatory learning activities, buzz sessions, brainstorming, lectures, and discussions. Participants were encouraged to engage and communicate with others about HIV prevention, treatment, and disclosure. These sessions focused on describing self-efficacy, self-esteem, autonomy, self-care, optimism, control over the future, stigma and discrimination issues, human and health rights, family and social relationships, and stress and righteous anger management.

Bryant et al. implemented an entertainment-education intervention where participants watched a single-session short film about HIV stigma and treatment [47]. The authors chose a film, “90 DAYS”, which focused on demonstrating the nuances of HIV stigma, such as diagnosis, disclosure, and partner rejection. The film aimed to illustrate stigma-related behavior and promote covert learning about HIV stigma.

Denison et al. implemented a peer mentoring intervention for the youth, a population distinct from adult populations described in the other included studies in this systematic review. Young individuals and adolescents living with HIV have unique requirements of support and resources to develop effective HIV self-management behaviors as they transition into adulthood. Self-management encompasses adherence to antiretroviral therapy (ART), practicing safer sex behaviors, and transitioning to adult care. In Denison’s study, the intervention arm included HIV-positive youth who had successfully transitioned to self-management. The intervention arm underwent two-week training and were assigned to be mentors to the study participants [48]. Mentorship activities entailed engaging others in group-based activities, such as discussing challenges, approaches, and ideas. Table 3 summarizes the interventions and their effectiveness in reducing HIV-related stigma and improving ART adherence.

**Table 3:** Intervention characteristics and study outcomes of all the studies eligible for inclusion published between January 1, 2013 to June 1, 2023 (data extracted from June 2023 to March 2024).

Citation	Intervention Type	Description			Effectiveness		
		Details	No. of Sessions	Session Duration	Stigma Reduction	ART/PrEP Adherence or Uptake	
						Assessment Method	Findings
Abbas et al. (2023)	Brief cognitive behavioral therapy	Psychoeducation; cognitive restructuring; stress management; skill training; relapse prevention	8	Not stated	The intervention had a large effect size on the social stigma, explaining approximately 78.7% variance in group differences post-intervention.	ART adherence was assessed using the GMAS	The intervention had a moderate effect size on the ART adherence, explaining approximately 50.3% variance in group differences post-intervention.
Bhatta and Liabsuetrakul (2017)	Empowerment intervention	Session content derived from social learning and action theory and empowerment principles of HIV prevention and treatment	6	1h 30 mins	At baseline, the mean values for stigma for intervention and control groups was 76.50 and 76.03 (23 item scale, with each item rated on 4-point agreement scale ranging from strongly disagree to strongly agree) respectively. At three months follow-up, the means changed to 39.41 and 72.91, respectively. The means changed to 38.26 and 73.03 at six months follow-up, respectively. The differences achieved statistical significance.	Single item (coded as yes or no) regarding ART adherence	A 1-unit increase in ART adherence was associated with a 1.3-unit decrease in internalized stigma.
Bogart et al. (2021)	Community-based cognitive behavioral therapy group intervention	Psychoeducation; skill training; acknowledging historical and current structural discrimination; functional chain analysis; session on ineffective coping; contextual understanding; sharing effective coping strategies; complexity of intersectional activities.	8	Not stated	In the intervention group, mean internalized stigma (1–5 point scale) reduced (SD) from 2.76 (1.20) at baseline to 2.38 (0.93) at 4 months and further to 2.14 (0.90) at 7 months. In the control group, internalized stigma reduced from 2.54 (0.88) at baseline to 2.38 (0.98) at 4 months but further increased to 2.65 (1.17) at 7 months. The differences between the two groups were statistically non-significant (Cohen’s *d* = 0.30).	Medication Event Monitoring System (MEMS) – ART adherence operationalized as the percentage of total scheduled doses taken.	In the intervention group, ART adherence (SD) improved from 94.61 (% of doses) (6.62) at baseline to 97.48 (5.42) at 4 months and further to 99.17 (1.51) at 7 months. In comparison, in the control group, ART adherence (SD) improved from 91.08 (8.97) at baseline to 94.57 (6.56) at 4 months and slightly reduced to 92.91 (16.78) at 7 months. The group differences were statistically significant (p = .02) with a small effect size (Cohen’s d = .26).
Bryant et al. (2023)	Entertainment-education	A short film embedded with social and health messages related to HIV status disclosure and medical adherence.	1	Not stated	No statistically significant difference in internalized stigma between the film (M = 2.95, SE = 0.14, n = 66) and brochure conditions (M = 2.93, SE = 0.13, n = 64), t(127) = -0.057, p = .955.	2-item scale assessing the likeliness of missing HIV medication over the next 30 days	No statistically significant difference in medical adherence intentions between the film (M = 2.49, SE = 0.18, n = 65) and brochure conditions (M = 2.56, SE = 0.08, n = 64).
Denison et al. (2020)	Youth peer mentoring	Involved HIV-positive youth mentors who had successfully transitioned to self-management; they underwent capacity building through 2-week training; youth mentors met with other HIV-positive youth to discuss challenges, approaches, and ideas.	Not stated	Not stated	In the control group, 45.2% of participants at baseline and 39.7% of participants at midline (6 months follow up) reported at least two of the three feelings associated with internalized stigma (shame, guilt, worthlessness) [OR:0.83, 95% CI:0.54,1.29]. In the intervention group, 50.4% of the participants at baseline and 25.4% at midline (6 months) reported at least two of the three feelings associated with internalized stigma [OR:0.39, 95% CI:0.21,0.7]. Time and group interactions were statistically significant.	2-item scale asking participants to indicate whether they missed ART at least once in the past three months and the number of consecutive days they missed ART in that period.	In the control group, 32.6% of participants at baseline and 33.9% at midline (6 months follow-up) reported an ART adherence treatment gap of more than 48 consecutive hours [OR:1.05, 95% CI: 0.68, 1.61]. In the intervention group, 45.3% of the participants at baseline and 34.4% at midline (6 months follow-up) reported an ART adherence treatment gap [OR 0.63, 95% CI: 0.35, 1.13].
Kalichman et al. (2019)	Behavioral self-regulation counseling	Grounded in theories of behavioral self-regulation and social cognition; derived from motivational interviewing framework and cognitive behavioral self-regulation; identification of barriers; addressing stigma concerns; subsequently weekly phone sessions; stigma components.	5	20-45 minutes	In the intervention group, 52% of the participants at baseline (compared to 64% of participants in control group) and 27% of the participants post-intervention (compared to 76% of participants in the control group) indicated they hid their ARVs in a secret place (OR 0.16, 95% CI,.035-.731, p = 0.01). In the intervention group, 20% of the participants at baseline (compared to 20% of participants in control group) and 36% of the participants post-intervention (compared to 4% of participants in the control group) indicated they remove ARVs out of their bottles so people will not know what they are (OR 17.33, 95% CI, 1.76-178.01, p < 0.01).	Visual analog scale.	In the intervention group, ART adherence at baseline (mean (SD)) was 87.0 (20.4), which increased to 96.3 (6.8) at follow-up. In the control group, ART adherence at baseline was 78.8 (28.5), which reduced to 76.8 (37.0) at follow-up. Group differences at follow-up were significant (OR 1.2, 95% CI, 1.05-1.38), p < .001.
Wagner et al. (2021)	Compassion-focused therapy principles and cognitive behavioral therapy strategies.	Experience sharing; group problem solving and role plays; personal goal setting; engaging in prevention advocacy; breaking down and coping with internalized HIV stigma; skills training; positive living health behaviors.	6 (group sessions)	Not stated	At the 5-month follow-up mark, the intervention group had significantly lower internalized stigma (mean (SD)) than the control group, 1.56 (0.61) vs 1.87 (0.73), p = 0.027.	Single-item scale asking participants to indicate the percentage of ART doses taken in the past month.	At 5 months follow-up, ART adherence (mean (SD)) remained similar between the intervention and control groups, 91.9% (8.9) vs. 92.7% (14.5), p = 0.74.
Eaton et al. (2023)	Brief counseling	First intervention group (Group 1) received a Jumpstart intervention comprising of motivational interviewing, client-centered counseling, and semi-structured interviewing styles; in the second intervention group (Group 2), participants received the Jumpstart intervention plus text messaging (asking participants if they needed help regarding their goals set during counseling), and the third intervention group (Group 3) received the Jumpstart intervention plus a phone call with similar intention as the text message in Group 2.	1	Jumpstart Intervention session of 45 minutes; text messaging or phone calling approximately 10–15 minutes.	No significant reduction in anticipated PrEP stigma; 17% of participants had complex barriers to PrEP adherence (including high levels of anticipated PrEP stigma), but this proportion increased to 19% post-intervention.	PrEP uptake assessed through self-reports (using PrEP in the past 4 months post-intervention) and biological testing.	In the control group, 24% reported PrEP uptake compared to 29.4% in Group 1, 34.0% in Group 2, and 37.3% in Group 3. However, a small effect size of 0.10 was reported.

GMAS – General medication adherence scale; OR – odds ratios; CI – confidence interval, SD – Standard Deviation, M—mean, SE – Standard Error.

### Interventions for PrEP adherence

The singular RCT examining PrEP adherence employed a unique 45-minute session intervention called project Jumpstart [51]. The session activities included motivational interviewing, client-centered counseling, and semi-structured interviewing. Four parallel arms were studied: control group, Jumpstart group, Jumpstart group (with additional 10–15 minute text message check-in), and Jumpstart group (with additional 10–15 minute text message and phone call check-in). Check-ins involved asking participants if they required assistance in achieving their PrEP-related goals established during the counseling session.

### Outcome measvurement instruments

All eight studies reported outcomes related to HIV-related stigma and ART/PrEP adherence or uptake using a wide range of measurement instruments.

For stigma reporting, three studies assessed HIV-related stigma using the Internalized AIDS-Related Stigma Scale [46,48,50]; one used the 40-item HIV Stigma Scale [44], one adopted a custom 23-item scale from previous research [45], one used the Negative Self-Image Scale [47], and others conceptualized HIV stigma based on previous research and developed custom 3-item scales, unique in each study [49,51].

Unlike HIV-related stigma, where most studies utilized previously validated measurement scales, ART/PrEP adherence outcomes were assessed using single or two-item scales formulated by the authors, lending heterogeneity in the outcome reporting (Table 3). Abbas et al. assessed ART adherence using a pre-and post-test design to evaluate the impact of cognitive behavioral therapy. Adherence was measured using the General Medication Adherence Scale (GMAS), which was originally developed in the Urdu language. The GMAS comprises 11 items, each with four possible response options, categorized into three domains: (1) non-adherence due to patient behavior (NAPB), (2) non-adherence due to other diseases and pill burdens (ADPB), and (3) non-adherence due to financial constraints (NAFC). Each item is scored on a scale from 0 to 3, with the total score determining the overall adherence level. Adherence is classified as high (30–33), good (27–29), partial (17–26), low (11–16), and poor (≤10). The GMAS demonstrates strong reliability, with an internal consistency of 0.80 and a test-retest reliability of 0.996[44]. Conversely, Bhatta and Liabsuetrakul assessed adherence to antiretroviral therapy (ART) as a dichotomous variable, categorizing participants’ responses as either “yes” or “no” based on their self-reported adherence. Participants were asked, “Have you been taking your medication?” at three key time points: baseline, three months after the first follow-up, and six months after the first follow-up. However, the study did not specify the exact criteria used to determine adherence, such as whether the classification was based on a specific number of missed doses or the participant’s subjective perception of their adherence [45].

Bogart et al. measured ART adherence using both electronic monitoring and self-report methods throughout the seven-month study. At baseline, participants completed a survey and were provided with a Medication Event Monitoring System (MEMS) bottle cap (AARDEX, Inc.), which electronically tracked adherence by recording the date and time of each bottle opening. The MEMS cap was assigned approximately two months before each cohort’s intervention start date to establish a valid pre-intervention baseline and minimize potential reactivity to the monitoring process. To ensure accurate tracking, research assistants helped participants transfer their medication into the MEMS-equipped bottle. When participants had multiple medications, the cap was used for the one with the most complex dosing schedule or, if all had the same schedule, the regimen’s base medication. Adherence data were downloaded at follow-up visits scheduled at two, four, five, and seven months post-baseline. At each follow-up visit, participants also completed a brief survey to account for instances where the MEMS cap may not have captured adherence accurately. This included reporting cases in which the bottle was opened without a dose being taken, doses were removed from an alternative source (e.g., a pillbox), or multiple doses were removed at once for later ingestion. These responses were used to adjust the MEMS data, refining the adherence assessment for greater accuracy. The primary adherence outcome derived from MEMS data was continuous adherence, calculated as the percentage of total scheduled doses taken during each one-month period of the study. In addition to electronic monitoring, self-reported adherence was assessed using a visual analog scale, where participants estimated the percentage of prescribed ART doses they had taken in the past month. This self-report measure was validated against objective indicators such as viral load and pill count, enhancing the reliability of adherence assessment [46].

Bryant et al. measured medication adherence intentions using a two-item Likert-type scale adapted from Stirratt et al. (2006). This scale assessed the likelihood of participants missing their HIV medication over the next 30 days due to nondisclosure of their HIV status. Responses ranged from “not at all likely” to “very likely,” capturing variations in adherence intentions. One of the items specifically asked, “Miss doses of your ART/ARV (HIV medication) in the next 30 days due to being with a sexual partner who does not know you are living with HIV.” The study employed a randomized controlled trial (RCT) with a pre- and post-test design to compare two conditions: (1) an active control condition, where participants received a standard-of-care brochure, and (2) the intervention condition, in which participants viewed the 90 DAYS film. Adherence intentions were measured before and after exposure to these conditions, allowing the researchers to assess the impact of the intervention on participants’ likelihood of missing doses due to nondisclosure [47].

In contrast, Denison et al. assessed ART adherence by measuring the presence of a treatment gap at two time points using two self-reported questions. Participants were asked: (1) “In the past three months, did you have a day when you did not take any ART drugs?” and (2) “What were the most days in a row that you missed swallowing your drugs in the past three months?” A binary adherence outcome was generated based on participants’ responses. Participants were classified as not having a treatment gap if they reported no missed full days of ART in the past three months or if they acknowledged missing ART but for only one day. In contrast, participants were considered to have an ART adherence treatment gap if they reported missing 48 consecutive hours or more within the past three months. If responses to both questions were missing, the adherence treatment gap outcome was also considered missing. This approach provided a structured way to assess adherence while emphasizing gaps in treatment that could impact clinical outcomes [48].

Kalichman et al. assessed ART adherence at baseline and follow-up using a reliable and validated rating scale that asked participants to estimate the percentage of their HIV medication doses taken in the past month. At baseline, adherence was measured using a visual analogue scale (VAS) graded along a continuum from 0% to 100% adherence. Participants were provided with the following instructions: “We know it is common for people to miss or skip taking doses of their ARVs (antiretrovirals). Think about the past month when answering this question. Think about what has happened over the past 30 days, that is, for one month before today. Using this rating scale, how much of your ARVs have you taken? You can answer anywhere from ‘None of my ARVs’ to ‘All of my ARVs’ or anywhere between that by marking a line. I am going to ask you to make a mark to show how much you HAVE taken in the past month: 0% means you have taken NONE of your ARVs in the past month, 50% means you have taken about HALF of your ARVs, and 100% means you have taken ALL of your ARVs and never missed a dose in the past month.” Participants were then instructed to mark their adherence level on the scale and state the corresponding percentage. For follow-up assessments conducted over the phone, the same adherence rating method was used, but without the visual scale. Instead, participants verbally reported their adherence percentage based on the same instructions and response stem. This method ensured consistency across assessments while allowing for adherence measurement in remote follow-ups [49]. Wagner et al. assessed ART adherence using a single-item self-report measure, in which participants rated the percentage of prescribed ART doses they had taken in the past month on a 0–100% scale. This measure served as an indicator of positive living behavior, providing a straightforward estimate of adherence based on participant recall [50].

Eaton et al. measured PrEP uptake through a combination of biological testing and self-report from follow-up assessments. Participants who reported any PrEP use within the four months post-intervention were asked to provide a urine sample for laboratory analysis using UrSure (UrSure, Inc., Boston, MA), a validated assay that detects the presence of tenofovir, the active ingredient in PrEP. Test results were interpreted dichotomously—indicating either the presence or absence of tenofovir—to objectively confirm self-reported PrEP adherence [51].

### Outcomes

Overall, of the seven studies examining ART adherence and stigma reduction in persons with HIV, three studies showed ART adherence, and five studies showed stigma reduction. The singular study that investigated PrEP adherence and stigma reduction showed significant PrEP adherence but not stigma reduction.

#### Outcomes for stigma reduction and ART adherence or uptake.

Two of the four RCTs that tested interventions with a cognitive-behavioral component found significant reductions in HIV-related stigma and improvements in ART adherence/uptake [44,49]. Table 3 provides specific statistical information as reported by individual studies.

Abbas et al. employed CBT and reported a substantial impact on social stigma. The post-intervention analysis revealed a substantial effect size, indicating that the intervention accounted for approximately 78.7% of the variance in group differences. This suggests that B-CBT significantly reduced social stigma among patients with HIV/AIDS (F(1,78) = 208.47, p < .000, η² = .787). Additionally, regarding ART adherence, the intervention explained about 50.3% of the variance in group differences post-intervention (F(1,78) = 24.75, p < .000, η² = .503). However, the actual difference in medical adherence, as reflected by GMAS scores, showed that CBT led to a 0.51% improvement in adherence levels in the experimental group (EXPg) compared to the waitlist control group (WLCg) [44]. Bogart et al. employed community-based CBT, noting shifts in internalized stigma - a decline from a mean of 2.76 (1.20) at baseline to 2.14 (0.90) (on a scale of 1–5) at seven months (Internalized Homophobia Scale-Revised, Scores 1–5) in the intervention group. In contrast, the control group’s internalized stigma declined initially but increased over time. The differences in internalized stigma reduction were statistically non-significant (Cohen’s d = 0.30).Electronically Monitored Adherence data showed a continuous trend in adherence over time for both the intervention and control groups, with a noticeable upward shift in adherence post-intervention, lasting 4–5 months. A repeated-measures regression revealed a marginally significant effect on adherence post-intervention, b (95% CI) = 9.24 (−0.55, 19.03), p = 0.06. This effect indicated that, during the three months following the intervention, participants in the intervention group had an adjusted adherence rate more than 9% higher, on average, than those in the control group, representing a medium effect size.

For self-reported adherence, repeated-measures regressions indicated that the intervention significantly improved self-reported adherence, b (95% CI) = 4.50 (0.70, 8.30), p = 0.02. While both groups showed an increase in the average percentage of doses taken, the intervention group showed a greater improvement relative to the control group, yielding a small effect size of 0.26.[46].

In the Behavioral Self-Regulation Counseling used by Kalichman et al., the intervention group exhibited reduction in participants’ inclination to hide antiretroviral (ARV) tablets significantly (52% at baseline to 27% post-intervention, p = .01). In the intervention group, 20% of the participants at baseline (compared to 20% of participants in control group) and 36% of the participants post-intervention (compared to 4% of participants in the control group) indicated they remove ARVs out of their bottles so people will not know what they are (OR 17.33, 95% CI, 1.76-178.01, p < 0.01). The practice of label removal from ARV bottles to maintain secrecy decreased from 8% at baseline to 4% post-intervention (p < .01) [49]. This group also showed a significant rise in ART adherence from 87.0 (20.4) at baseline to 96.3 (6.8) (self-reported scale, 0–100% adherence score) at 5-week follow-up. In contrast, adherence declined in the control from 78.8 (28.5) to 76.8 (37.0). These group differences were significant (OR 1.2, 95% CI, 1.05-1.38, p < .001), supporting the efficacy of the intervention. The difference (between behavioral self-regulation and controls) observed was both statistically significant and clinically meaningful, with Cohen’s d = 0.69. Post-hoc analyses within the groups revealed that the counseling group experienced a significant improvement in adherence from baseline to follow-up, t(21) = 2.21, p = 0.03, Cohen’s d = 0.58. In contrast, the control group’s adherence remained unchanged, t(22) = 0.14, p = 0.89, Cohen’s d = 0.06. Further analysis of intervention dosage revealed that four participants received one or two counseling sessions, 14 received three or four sessions, and seven participants completed all five sessions. The most notable improvement in adherence was seen in those who received three or four counseling sessions [49]. Wagner et al.’s 2021 study, emphasizing Compassion-Focused Therapy principles and CBT, found that the intervention group exhibited significantly lower internalized stigma (Internalized AIDS-Related Stigma Scale Score 1–5) compared to the control group at the 5-month follow-up (1.56 vs. 1.87, p = 0.027) [50,53]. However, no significant differences in ART adherence/uptake were observed (91.9% vs. 92.7%, p = 0.74).

These studies unveil the multifaceted impact of stigma reduction interventions, providing a nuanced understanding of their effects on social stigma and ART adherence across diverse methodologies. The variable findings of the four RCTs above can be attributed to heterogeneous treatment protocols, the use of different treatment measurements for stigma/adherence, and variable risk of bias. Abbas et al. (2023), Bogart et al. (2021), and Wagner et al. (2021) had a high risk of bias [44,46,50], whereas Kalichman et al. (2019) only had a few concerns [49].

Other interventions that showed effectiveness in reducing HIV-related stigma and simultaneously improving adherence to ART include empowerment intervention [45], youth peer mentoring intervention [48], and entertainment education [47].

Bhatta and Liabsuetrakul’s 2017 study on empowerment as a stigma reduction intervention reported the initial mean scores at baseline for both intervention and control groups were 76.50 and 76.03, respectively. Notably, significant shifts occurred at three- and six-month follow-ups (intervention group means dropped to 39.41 and 38.26; control group to 72.91 and 73.03, respectively) [45]. This divergence achieved statistical significance. Regarding ART adherence, the intervention demonstrated a noteworthy association—each 1-unit increase in ART adherence corresponded to a 1.3-unit decrease in internalized stigma. Denison et al. utilized youth peer mentoring as a stigma reduction intervention. The findings revealed a substantial reduction in internalized stigma in the intervention group (50.4% at baseline to 25.4% at six months), with statistically significant time and group interactions (OR: 0.39, 95% CI: 0.21, 0.7) [48]. In contrast, the control group exhibited a milder reduction (45.2% baseline to 39.7% at six months), with a less significant impact (OR: 0.83, 95% CI: 0.54, 1.29). The intervention group demonstrated adherence treatment time gap compared to the control group (OR: 0.63, 95% CI: 0.35, 1.13).

Bryant et al.‘s 2023 study used entertainment education (short film as stigma reduction intervention) and showed subtle differences. Post-intervention mean differences for stigma reduction were 2.95 in the intervention group and 2.93 in the control group (p = .955). Post-intervention mean differences for ART/PrEP adherence/uptake were 2.49 in the intervention group and 2.56 in the control group (p = .782) [47].

#### Outcomes for PrEP adherence.

Eaton et al. reported that the Jumpstart program did not significantly reduce anticipated PrEP stigma but increased PrEP adherence [51]. After a Brief Counseling intervention, 17% of participants exhibited complex barriers to PrEP adherence, including elevated levels of anticipated PrEP stigma, a proportion that marginally increased to 19% post-intervention. Discernible differences in PrEP adherence/uptake emerged among the groups. In the control group, 24% reported PrEP uptake; this increased in the intervention groups (29.4% in group 1, 34.0% in group 2, and 37.3% in group 3) and a 43% higher likelihood of uptake in the Jumpstart text/phone call arm compared to the control condition. Despite these variable increases in uptake, the effect size remained very weak (Cohen’s d 0.10), indicating a limited impact of the Brief Counseling stigma reduction intervention on the overall prevalence of PrEP uptake across the groups.

### Quality assessment

Table 4 summarizes the quality assessment of each study. The quality of the RCTs ranged between 58.33% and 91.67%. Six of the eight RCTs had one or two domains with a high risk of bias. In these studies, Domain 2(a) (effect of assignment to intervention) and Domain 2(b) (effect of adhering to intervention) had a high risk of bias since most of the RCTs were open-label, meaning the participants, intervention deliverers, and outcome assessors were aware of the assignment of participants to control and intervention groups. However, the remaining domains, including randomization and handling of missing outcome data, had a low risk of bias.

**Table 4:** Risk of bias assessment findings of all the studies eligible for inclusion published between January 1, 2013 to June 1, 2023 (data extracted from June 2023 to March 2024).

Citation	Domain 1(a)	Domain 1(b)	Domain 2(a) (effect of assignment to intervention)	Domain 2(b) (effect of adhering to intervention)	Domain 3	Domain 4	Domain 5	Overall Judgment (% score)
Abbas et al. (2023)	Low risk of bias (2)	NA	High risk of bias (0)	High risk of bias (0)	Low risk of bias (2)	Low risk of bias (2)	Low risk of bias (2)	High risk of bias (66.67%)
Bhatta and Liabsuetrakul (2017)	Low risk of bias (2)	NA	Some concerns (1)	Low risk of bias (2)	Low risk of bias (2)	Low risk of bias (2)	Low risk of bias (2)	Some concerns (91.67%)
Bogart et al. (2021)	Low risk of bias (2)	NA	High risk of bias (0)	High risk of bias (0)	Low risk of bias (2)	Low risk of bias (2)	Low risk of bias (2)	High risk of bias (66.67%)
Bryant et al. (2023)	Some concerns (1)	NA	High risk of bias (0)	High risk of bias (0)	Low risk of bias (2)	Low risk of bias (2)	Low risk of bias (2)	High risk of bias (58.33%)
Denison et al. (2020)	Low risk of bias (2)	NA	High risk of bias (0)	High risk of bias (0)	Low risk of bias (2)	Low risk of bias (2)	Low risk of bias (2)	High risk of bias (66.67%)
Kalichman et al. (2019)	Low risk of bias (2)	NA	Some concerns (1)	Some concerns (1)	Low risk of bias (2)	Low risk of bias (2)	Low risk of bias (2)	Some concerns (83.33%)
Wagner et al. (2021)	Low risk of bias (2)	Low risk of bias (2)	High risk of bias (0)	Some concerns (1)	Low risk of bias (2)	Low risk of bias (2)	Low risk of bias (2)	High risk of bias (78.57%)
Eaton et al. (2023)	Low risk of bias (2)	NA	High risk of bias (0)	High risk of bias (0)	Low risk of bias (2)	Low risk of bias (2)	Low risk of bias (2)	High risk of bias (66.67%)

The risk of bias assessment tool (RoB) can be accessed here: https://drive.google.com/file/d/1a7HtfocC74obX1NfW5YnKS0-4pEmis92/view

Overall judgment was presented as a percentage. Each domain was numerically scored as follows: (a) High risk of bias – (0), (b) some concerns – (1), and (c) low risk of bias – (2). An RCT (non-cluster) can have a maximum score (summation of domain scores) of 12 and a minimum score of 0, whereas a cluster RCT can have a maximum score of 14 and a minimum score of 0. Overall judgment was calculated as the percentage of the study’s score divided by the maximum possible score.

## Discussion

This systematic review aimed to identify effective interventions that reduce HIV-related stigma while simultaneously improving adherence to ART or PrEP. The findings revealed promising approaches that combine psychoeducation, skills training, and patient-centered support [44,49]. These interventions empower individuals by fostering knowledge, coping mechanisms, and a sense of community. However, the limitations in current research designs, the lack of universal standardized approaches, and a narrow focus on internalized stigma within interventions highlight critical gaps in our understanding.

The pioneering work on HIV-related stigma started many decades ago, with Goffman describing stigma as “a *dynamic process of devaluation that ‘significantly discredits’ an individual in the eyes of others*” [14]. Since then, many models to understand stigma have been published [13,54–58]. Earnshaw and Chaudoir proposed a comprehensive framework to provide an understanding of HIV-related stigma [15]. Building on previously proposed models, they acknowledge that “*a stigma is a “mark” or aspect of the self that is socially devalued*.” Keeping this as the core tenet, they argue that in addition to social processes, individual domains (such as mental illness, sexual orientation, racism, etc.) should also be considered to understand the outcomes of stigma. This framework describes the dichotomy of mechanisms between people who are HIV-negative and PLWH and how the outcomes of each side impact the other. It also highlights the variability in the outcomes of stigma among different individuals and communities. HIV-negative individuals who do not possess the so-called “devalued attribute” feel threatened due to prejudice, stereotypes, and discrimination, leading to social distancing and impact on policy/research. PLWH may internalize this devalued attribute feeling (internalized stigma), leading to experiences such as physical violence, social rejection (enacted stigma), or fear of future rejection (anticipated stigma). Internalized stigma also largely This framework provides a starting point for considering the “*who, how,* and *what”* components of research study designs that aim to investigate strategies addressing stigma.

The reviewed studies identified interventions with the potential to achieve the dual goals of reducing stigma and improving adherence. Approaches that combine psychoeducation, skills training, and patient-centered support appear particularly effective. These interventions likely empower individuals by increasing knowledge about HIV, building coping mechanisms to manage stigma, and fostering a sense of community through shared experiences. Notably, interventions lacking sustained engagement, such as single-session entertainment-education approaches, demonstrated lower efficacy [47]. This highlights the importance of ongoing support and interactive elements in intervention design.

Most included studies predominantly focused on internalized stigma, with only one study each looking at anticipated and social stigma [44,51]. This approach is concerning, as the Earnshaw-Chaudoir framework suggests that addressing all forms of stigma is crucial for optimal outcomes [15]. Interventions solely targeting internalized stigma neglect the fear of discrimination that hinders healthcare access, ultimately impacting adherence. For instance, anticipated stigma causes individuals to fear discrimination, which might lead them to avoid seeking testing or initiating treatment altogether. Balancing measures are needed in designing studies to address various components of stigma experienced by an individual to obtain a more precise measure of this construct. This could be done by using more comprehensive instruments with validity evidence supporting the investigation of multidimensional nature of stigma experienced by a single individual.

This systematic review has helped identify critical areas for future research. First, robust studies with larger and diverse populations are needed. The effectiveness of psychoeducation, skills training, and patient-centered approaches merits their incorporation in future double blinded RCTs, while considering the tenets of Earnshaw-Chaudoir framework. Designing double-blinded randomized studies for stigma-reduction interventions to improve ART adherence or uptake is challenging due to the subjective nature of stigma and the practical difficulties of blinding in such sensitive contexts. However, several approaches can help overcome these challenges. One strategy is blinding the assessment of outcomes, where assessors remain unaware of the intervention group to reduce bias in outcome measurement, such as adherence rates or stigma reduction. Using a placebo intervention in the control group can also isolate the effects of the stigma-reduction intervention by ensuring that both groups are receiving support related to ART adherence, even if the content differs. Digital or automated interventions, such as apps or online platforms, can be used to standardize the delivery of content and enhance blinding, as they remove human interaction from the process. Objective measures, such as pharmacy refill records or session attendance, alongside survey-based outcomes, can also reduce biases inherent in self-reporting. Finally, ensuring proper randomization, counterbalancing, and ethical considerations is crucial to minimize harm and bias while still producing reliable, rigorous results in stigma-reduction interventions. The limited success of single-session interventions signals that populations need longitudinal support and engagement. Second, interventions should address not only internalized stigma but also anticipated and enacted stigma. In addition, addressing intersectional stigma in HIV programs is essential to ending the epidemic. However, rather than isolating intersectional stigma, we must integrate it with other forms of stigma to address the broader, interconnected oppressive systems such as structural racism, ageism, sexism, ableism, homophobia, transphobia, xenophobia, etc.[46,50,53,59,60]. Current approaches tend to emphasize the multiple stigmatized identities rather than the historical legacy of structural oppression that drives HIV inequities. Third, HIV-negative individuals have a crucial in mitigating societal stigma; as such researchers must identify practical ways to address stigma through training, allyship, trauma-informed care, etc. Fourth, interventions targeting healthcare providers (e.g., education, quality improvement initiatives, and patient safety programs embedded within health policy) can help dismantle prejudice and discrimination, creating a more supportive clinical culture for adherence.

Addressing stigma through multifarious interventions can improve uptake and adherence to ART and PrEP. This systematic review highlights that no single methodology or instrument provides a comprehensive measure of assessing stigma reduction. Social sciences research should also focus on head-to-head comparisons of which methodologies or combinations thereof could be more effective than others. The mechanisms that directly or indirectly link to stigma reduction and medication adherence through various interventions are numerous – reduced fear of social discrimination among PLWH can encourage individuals to seek testing and initiate treatment. Skills training for medication adherence and coping with adverse effects can empower individuals to manage their treatment. A comprehensive approach that tackles both stigma and treatment adherence is crucial for achieving viral suppression and curbing viral transmission.

### Limitations

Certain limitations of this study merit discussion. First, most studies were open-label which introduces bias. Second, significant heterogeneity exists in methodologies, making head-to-head comparisons challenging to isolate the most effective intervention. Future research should prioritize robust RCTs with larger, more diverse populations and standardized outcomes. Double-blinded designs with adequate sample size, participant allocation, and blinding can minimize bias. Third, the low number of studies with small sample sizes representing limited geographical cohorts makes the generalizability of the findings challenging. The socio-cultural influences that accompany geographical diversity are crucial to the understanding of multidimensional and complex nature of HIV related stigma. Hence, studies need to be conducted in more diverse regions. Fourth, six of the eight included studies had a high risk of bias. This, combined with the utilization of varied scales and methods, calls for standardized measures in assessing ART/PrEP adherence outcomes for future research to enhance comparability and interpretation of findings.

## Conclusion

This systematic review underscores the multifaceted nature of stigma reduction interventions and emphasizes the need for tailored approaches to address diverse populations and contexts. While cognitive-behavioral interventions show promise, the variability in outcomes across different methodologies and interventions informs the need for continued research, methodological refinement, and the development of standardized measures to assess medication adherence. Concurrently, it is important to design studies integrating intersectional stigma with other forms of stigma. As the field progresses, embracing a comprehensive and individualized approach will be paramount in designing interventions that not only reduce stigma but also enhance adherence to therapy.

## Supporting information

S1 File Search strategy. (DOCX)

S2 File Table of all included and excluded studies. (XLSX)
