# Supplementary material for: Interventions for stigma reduction in HIV treatment and prevention designed to enhance antiretroviral uptake and adherence: A systematic review
Source: PLOS Glob Public Health. 2025 May 12;5(5):e0004604. doi: 10.1371/journal.pgph.0004604 (PMC12068570; doi:10.1371/journal.pgph.0004604)
Supplement: S1 File — (DOCX) [file pgph.0004604.s001.docx]

**S1 File:** Search Strategy/keywords used for multiple database inquiry

**Key Concepts**

1. **Stigma**

*Keywords*: stigma*, shame, internalized stigma

*MeSH*: "Social Stigma"[Mesh]

1. **HIV**

*Keywords*: HIV, human immunodeficiency virus, HIV prevention, HIV treatment, HIV/AIDS, ART, ARVs, PrEP

*MeSH*: "HIV"[Mesh], "Antiretroviral Therapy, Highly Active"[Mesh], "Pre-Exposure Prophylaxis"[Mesh]

1. **Adherence**

*Keywords*: Uptake, adherence, medication adherence, medication compliance, drug compliance,

*MeSH*: "Medication Adherence"[Mesh]

**Search Strategy**

| **Search Round** | **Keywords** | **Results Before Limiters** | **Results After Limiters** | |
| --- | --- | --- | --- | --- |
| #1 | "Social Stigma"[Mesh] OR Stigma*[tiab] OR shame[tiab] OR “internalized stigma”[tiab] | 60.45K | RCT | 1.28K |
|  |  |  | English | 1.27K |
| #2 | "Pre-Exposure Prophylaxis"[Mesh] OR "HIV"[Mesh] OR "Antiretroviral Therapy, Highly Active"[Mesh] OR HIV[tiab] OR “human immunodeficiency virus”[tiab] OR “HIV prevention”[tiab] OR “HIV treatment”[tiab] OR HIV/AIDS[tiab] OR ART[tiab] OR ARVs[tiab] OR PrEP[tiab] | 522.03K | RCT | 11.96K |
|  |  |  | English | 11.84K |
| #3 | "Medication Adherence"[Mesh] OR uptake[tiab] OR adherence[tiab] OR “medication adherence”[tiab] OR “medication compliance”[tiab] OR “drug compliance”[tiab] | 593.05K | RCT | 19.96K |
|  |  |  | English | 19.69K |
| #4 | #1 AND #2 AND #3 | 2.32K | RCT | 111 |
|  |  |  | English | 111 |
